# Eyeless Mexican Cavefish Save Energy by Eliminating the Circadian Rhythm in Metabolism

**DOI:** 10.1371/journal.pone.0107877

**Published:** 2014-09-24

**Authors:** Damian Moran, Rowan Softley, Eric J. Warrant

**Affiliations:** Department of Biology, Lund University, Lund, Sweden; Simon Fraser University, Canada

## Abstract

The eyed surface form and eyeless cave form of the Mexican tetra *Astyanax mexicanus* experience stark differences in the daily periodicities of light, food and predation, factors which are likely to have a profound influence on metabolism. We measured the metabolic rate of Pachón cave and surface fish at a fixed swimming speed under light/dark and constant dark photoperiods. In constant darkness surface forms exhibited a circadian rhythm in metabolism with an increase in oxygen demand during the subjective daytime, whereas cave forms did not. The lack of circadian rhythm in metabolism leads to a 27% energy savings for Pachón cave fish compared to surface fish when comparing both forms in their natural photoperiods. When surface forms were tested under constant dark conditions they expended 38% more energy than cave forms under equivalent conditions. Elimination of the circadian rhythm in metabolism may be a general feature of animals that live in perpetually dark food-limited environments such as caves or the deep sea.

## Introduction

The Mexican tetra (*Astyanax mexicanus*) is useful for studying evolutionary adaptation as it comprises two distinct morphs: a fully-eyed surface-dwelling form and multiple cave-dwelling forms (with partial to total visual system degeneration) descended from surface ancestors. Fish from the Pachón caves in northeastern Mexico are the most troglomorphic (cave-adapted) and studied cave form (this morph has evolved independently on a number of occasions [1]), and exhibit common adaptations to life underground, including a loss in visual capacity and pigmentation, an increased reliance on taste [2] and mechanosensation [3] for navigation and foraging, and improved benthic foraging behaviour to facilitate detrital food location [4]. The loss of visual capacity leaves the cave forms extremely vulnerable to avian or piscine predators in overground rivers, which along with geological features strongly restricts their distribution to caves. Seasonal flooding can wash surface fish into caves [5], however, the condition of these individuals tends to be poor [6], which likely reflects a lack of benthic (river bottom) foraging capacity [4], reduced food availability in the caves (for at least part of the year [7]), and possibly a higher metabolic rate compared to cave adapted conspecifics [6], [8]. While there is a reasonably good understanding of the sensory and foraging adaptations that have allowed cave forms of *A. mexicanus* to survive underground [4], [8]–[10], much less is known about the degree to which their physiology and energy expenditure has changed. Hüppop [11] reported that Pachón morphs had a 27% reduced resting metabolic rate compared to surface morphs, however, reanalysis of this study later suggested there was no difference when fat content was taken into account [12]. In contrast, Salin et al [13] reported a 50% reduced metabolic rate of the Pachón morph compared to the surface morph. The discrepancy between these studies has made it difficult to evaluate the importance of metabolic rate as a factor in the physiological adaptation of *A. mexicanus* to life underground. Resolving this issue is particularly timely given the rising importance of this species as a model in evolutionary developmental biology [14]–[17], and also because energy saving may have been a significant factor in the reduction and loss of the visual system in cave morphs [18].

One shortcoming of earlier respirometric studies comparing cave and surface *A. mexicanus* is their relatively short duration (3–24 h) and the fact that they have not tested the effect of light or photoperiod on metabolism [11], [13]. These factors are relevant given that it can take fish more than a day to become acclimated to respiratory chambers [19], and also because a recent study has shown that oscillation of clock genes in cave forms of *A. mexicanus* are substantially altered [20], suggesting that clock-dependent physiological functions are also likely to differ between surface and cave forms throughout a 24 h cycle. Studies of both mammals and fish have shown that the metabolism follows an autonomous circadian rhythm independent of the metabolic costs imposed by the daily variation in movement and feeding [21]–[27]. The mechanisms by which molecular circadian oscillators are coupled to metabolism are becoming well understood in mammals [27], however, little is known about the relationship between metabolism and internal clocks in lower vertebrates such as fish. Fish appear to have a more complicated circadian clock arrangement than mammals (the fish circadian system is thought to be a network of interconnected circadian units, whereas mammals have a master clock located in the brain [28]), with a greater degree of clock decentralization [29]. Fish tend to exhibit a wider intra- and interspecies variability in the design and function of circadian systems compared to mammals, which is possibly due to coexisting or competing central and peripheral oscillators and a large number of circadian clock genes in these lower vertebrates [28], [30]. Despite the challenges that fish present in terms of understanding circadian biology, one experimental advantage these animals have is the ability to effectively set a single activity level by controlling swimming speed in a flume respirometer. Providing the flume current velocity is not too intense and the fish can maintain the swimming speed long-term, the minimisation of circadian variations in activity allows researchers to investigate the endogenous metabolic rhythm [21], [26]. Comparable conditions are difficult to achieve in other vertebrates, and while constant routine protocols have been developed to help isolate physiological circadian rhythms in mammals, these protocols tend to be restricted to human studies [31]. Consequently, endogenous rhythms in metabolic rate are a poorly explored phenomenon in circadian biology.

The lack of external cues for light/dark periodicity in caves coupled with a low pressure to synchronise physiology with food or predator abundance may have altered the circadian rhythm in metabolism of cave dwelling *A. mexicanus*, but this has not been investigated. The alteration of the molecular clock and lack of an endogenous activity pattern in Pachón *A. mexicanus* [20] suggests that this morph lacks a circardian rhythm in metabolism. We set out to test whether there are differences in the circadian rhythm in metabolic rate of surface and Pachón *A. mexicanus* by measuring oxygen consumption under light/dark and constant dark conditions over several days. The fish were tested in a flume that imposed a set swimming speed and allowed for the measurement of circadian metabolism by removing the masking effects of activity level and feeding. The link between metabolic rate and circadian rhythm has not before been considered in the context of evolution in food- and light-limited environments, however, this coupling may be of considerable importance for animals that diversified into perpetually dark environments such as caves and the deep ocean.

## Methods

### Experimental animals and circadian respirometry

Animal ethics permission for this study was granted by the Swedish Board of Agriculture (project approval number M119-10). *Astyanax mexicanus* from the Pachón cave population and pure surface forms derived from the Rió Tampaón stock were obtained from the Borowsky Lab at New York University in June 2010 (holding and breeding conditions described in [17]). Experiments described in this study were performed between February-November 2012. The fish were approximately 2 years old when tested, and were maintained in 100 L tanks at 20±0.5°C. The individual weight of fish used in the trial varied from 1.85–8.45 g, and standard length varied from 42–68 mm (Table 1). Fluorescent room lighting provided a 12:12 h light:dark cycle with 30 min long artificial sunrises and sunsets. Fish were fed daily between 09:00 and 16:00 on a combination of commercially available dry flake diet, frozen mosquito larvae and liver paste. Feeding times varied day to day to minimize the possibility of this being an entrainable zeitgeber.

**Table 1:** Summary data for metabolic rate of surface and Pachón *A. mexicanus* tested under two photoperiods.

	12:12 light:dark		24 dark		Significance test^a^
	Pachón	surface	Pachón	surface	
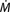 O_2min_, mg O_2_ h^−1^ g^−1^; mean±SD (range)	0.193±0.033 (0.154 to 0.243)	0.218±0.043 (0.145 to 0.284)	0.173±0.070* (0.093 to 0.178)	0.271±0.089* (0.261 to 0.443)	H_3,31_ = 9.152, *P* = 0.027
Scaled 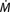 O_2min_, mg O_2_ h^−1^ 5 g^−1^ (mean±SD)	0.939±0.143	1.026±0.165	0.832±0.346	1.251±0.351	H_3,31_ = 5.373, *P* = 0.146
Scaled 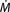 O_2mean_, mg O_2_ h^−1^ 5 g^−1^ (mean±SD)	1.171±0.159	1.342±0.333	0.991±0.291*	1.603±0.440*	H_3,31_ = 8.304, *P* = 0.040
bScaled daytime 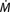 O_2mean_, mg O_2_ h^−1^ 5 g^−1^ (mean±SD)	1.194±0.151	1.494±0.497	0.999±0.296*	1.704±0.472*	H_3,31_ = 9.698, *P* = 0.021
bScaled nighttime 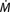 O_2mean_, mg O_2_ h^−1^ 5 g^−1^ (mean±SD)	1.159±0.174	1.215±0.211	0.984±0.289	1.512±0.436	H_3,31_ = 7.051, *P* = 0.070
bDaytime total O_2_:Nighttime total O_2_ (mean±SD)	1.050±0.098	1.267±0.252*	1.049±0.053	1.150±0.126*	t-test, deviation from 1
Daily metabolic peak, time of day (mean±SD)	16:30±2:12	17:13±2:07	14:04±2:37	11:38±4:02	
Scaled daily O_2_ uptake, mg O_2_ day^−1^ 5 g^−1^ (mean±SD)	28.23±3.81	32.51±8.23	23.79±7.01*	38.60±10.70*	H_3,31_ = 8.413, *P* = 0.038
cScaled energy expenditure, J day^−1^ 5 g^−1^	384	442	323	525	
Individual body mass, grams; mean±SD (range)	4.50±1.65 (3.30 to 8.45)	3.76±2.08 (1.85 to 7.36)	3.58±1.14 (2.02 to 5.20)	3.22±1.44 (1.87 to 5.38)	*F*_3,27_ = 0.914, *P* = 0.447
Standard length, mm; mean±SD (range)	54±6 (48–68)	54±7 (47–67)	51±5 (43–57)	52±8 (42–65)	*F*_3,27_ = 0.445, *P* = 0.723
Number of individuals tested	9	8	6	8	

*denotes difference between treatments and morphs at *P*<0.05, or significant (*P*<0.05) deviation from 1 for day:night total O_2_ consumption

a Significance test either Kruskall-Wallis (H-statistic), ANOVA (*F*-statistic), or t-test

b Subjective daytime and nighttime for continuous dark conditions

c Based on an oxyenergetic equivalent of 13·59 kJ g^−1^ O_2_ [36].

**Figure 1:**
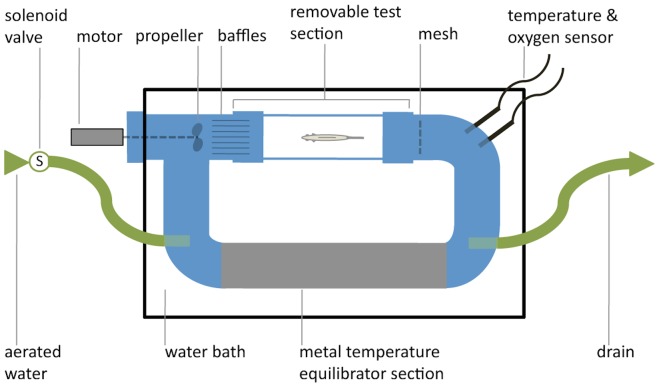
Schematic of the respirometer used in the study (not to scale).

The oxygen consumption rate of the fish was quantified using custom-built 1.6 L Brett-style tunnel flume respirometers with automated flushing (for intermittent flow respirometry) and maintained at 20±0.25°C (Figure 1). The fish were placed in a 400 ml test section (5×20 cm) with a laminar water flow provided by a propeller powered by an external motor. Fish were not fed during respirometric testing due to the significant impact the specific dynamic action (i.e. digestion and assimilation) would have had on daily oxygen consumption patterns. A four-channel oxygen optode and temperature sensor system (FireSting O2 system, PyroScience, Aachen, Germany) was used to measure the oxygen concentration in four respirometers. Background bacterial respiration was quantified in a respirometer without fish and subtracted from the oxygen depletion rates of the three other respirometers containing fish. The oxygen decrease in the fish-free respirometers was less than 5% of that recorded in the fish respirometers. Pilot testing of both fish types showed that 10 cm s^−1^ was the lowest flow velocity that could reliably elicit regular swimming behaviour from surface fish. At a lower flow velocity (2 cm s^−1^) surface fish exhibited varying degrees of agitation (repeated turning behaviour), even after an acclimation period of 4 hours. In the field surface fish are found swimming against the current to maintain their position the stream [8], which likely explains why these fish exhibited more constant swimming behaviour at a low to moderate current velocity (10 cm s^−1^) compared to a very low velocity (2 cm s^−1^). Other studies working with different species have also reported that fish held under low to moderate water flow rates exhibit less spontaneous activity and are less stressed (and hence less metabolically active) compared to being held in stationary water conditions [32]–[34].

A single oxygen consumption measurement cycle consisted of 15 min closed respirometry followed by a 5 min flush cycle. The oxygen concentration inside the respirometer was measured every 15 sec, and the oxygen saturation levels were above 80% at all times, avoiding hypoxic stress. The oxygen decrease over the 15 min closed period was between 3–7% oxygen saturation. Oxygen consumption rate was calculated using the linear portion of the oxygen concentration profiles (the middle 13 min of the 15 min measurement period). The linearity (coefficient of determination, *R* ^2^) of oxygen decrease during the aforementioned 13 min integration period was 0.94–0.99. Oxygen consumption rate was calculated using equation 2 of Clark et al [35].

Oxygen consumption was quantified for 6–9 surface and cave fish under two experimental conditions: (*a*) the 12:12 LD cycle of the aquaria room (7 consecutive days without feeding at a swim speed of 10 cm s^−1^ water velocity); (*b*) constant darkness (7 consecutive days without feeding at a moderate water velocity of 10 cm s^−1^, and no adjustment period from the 12:12 LD photoperiod of the aquarium holding conditions). A flow rate of 10 cm s^−1^ is equivalent to relative swimming speeds of approximately 1.5–2.4 body lengths s^−1^ (BL s^−1^). The length of time the fish were tested was sufficient to allow for repeated measurements of individual diel or circadian patterns in metabolism while avoiding any significant degree of starvation (no substantial decrease in metabolic rate was observed after adjustment to confinement, whereas other studies of starvation in this species have observed significant decreases in metabolic rate under starvation conditions [11], [13]). The fish were not fed the day prior to testing, so that they were at least 40 h post-ingestion at the time of experimentation. Experiments started between 10:00 and 16:00. The animals were weighed and their lengths measured at the end of the testing period. The 24 D photoperiod measurements were made by covering the clear test portion of the respirometer with black plastic, which was sufficient to prevent any light escaping (determined by placing a flashlight in the test section). The respirometers were located in a dedicated wet lab, and apart from the experimenters, no other people entered the room, which ensured minimal disturbance to the fish. The position of the respirometers was such that the fish being tested could not view other fish, and a curtain prevented incidental observation of people. Under 12:12 LD fish in the respirometers were visually inspected twice per day (once between 08:30–10:00 and once between 16:00–17:30), and the oxygen consumption traces were checked multiple times per day from a remote computer. Under 24D oxygen consumption traces were used to ascertain the status of the fish. There were no significant differences between treatments in either individual mean weight (ANOVA, *F*_3,27_ = 0.914, P = 0.447) or standard length (ANOVA, *F*_3,27_ = 0.445, P = 0.723). The data used in this study is available from the Dryad Repository (*accession number to be included following review and assignment of manuscript ID*).

### Metabolic rate definitions

Both a mean and minimum oxygen consumption rate (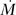
O_2mean_ and 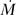
O_2min_, respectively) were calculated as these measures were used in two other studies of *A. mexicanus* [11], [13]. The 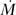
O_2mean_ is equivalent to Routine Metabolic Rate and represents the metabolism of a resting, unfed fish that shows frequent spontaneous variations in energy expenditure [36]. This was the closest approximation of *in situ* energy expenditure given the limitations imposed by the use of a respirometry chamber. The term 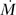
O_2min_ is a measure of minimal metabolism, but as we were not able to test our surface fish in a state where they were expending zero energy on swimming, we could not properly use the term Standard Metabolic Rate (SMR, equivalent to Basal Metabolic Rate in mammalian physiology). The value of 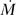
O_2min_ in the present study is not as an absolute measure of basal metabolism, but rather it offers a controlled comparison of minimal metabolism (as the activity costs were the same for both morphs and it gives a comparable measure of metabolic rate from morphs with very different patterns in circadian respiration rate). Should 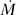
O_2min_ differ substantially, then it is likely there is a significant divergence in the underlying energetic physiology of each morph. We used the lowest observed oxygen consumption rate to define 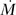
O_2min_ as this is a common approach in studies of minimal fish metabolism [35] and two other studies of *A. mexicanus* had used this definition [11], [13]. We heeded the advice of Herrmann & Anders [34] by using the mean value of the lowest 10% of the data to derive a repeatable minimal metabolism result (whereas taking the absolute lowest value can lead to spurious results in large data sets such as ours).

### Data analysis

The variables of interest for analysis were 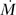
O_2mean_ (mean 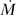
O_2_ for duration of testing), 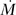
O_2min_ (lowest decile 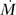
O_2_ during testing), nighttime and daytime mean 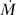
O_2_ (subjective daytime was used for the 24D treatment), and total oxygen consumed per day (area under the curve calculated using the trapezoid approach). The 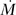
O_2min_ data for each morph under their natural photoperiod was plotted and a power function fitted to describe the allometric scaling relationship (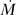
O_2min_ = 0.224 x body weight^0.862^, r = 0.731). The oxygen consumption data for each individual was then allometrically scaled to a standardised body mass (5 g) to account for the effect of body size. Mean metabolic rates were analysed per individual and time period, and a group mean and standard deviation generated. Statistically significant differences among treatments and morphs were analysed using Kruskall-Wallis tests together with Dunn's post hoc in Statistica 9.0 (Statsoft, Tulsa, U.S.A). Parametric testing was not appropriate given a strong positive correlation between mean values and variance. The oxygen consumption data were plotted according to the number of days elapsed since the experiments began to investigate any changes associated with habituation or starvation. The initial 36 hours of respirometry data were not included in subsequent analyses due to an obvious period of elevated oxygen consumption associated with acclimation to the respirometer. To determine whether the amplitude in the circadian rhythm in metabolism changed over time, the total daytime oxygen consumption was compared to the following nighttime total oxygen consumption, and the day:night O_2_ demand plotted against time. For the 24D photoperiod treatment the subjective daytime and nighttime were used. Single sample t-tests were used to test whether mean day:night O_2_ demand deviated from 1. The daily variation in mean 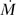
O_2_ was plotted (divided into 1 hour data bins) to inspect the circadian patterns in 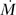
O_2_. Statistical differences in hourly-differentiated plots were investigated using Mann-Whitney U tests.

## Results

### Oxygen consumption patterns over a week

**Figure 2:**
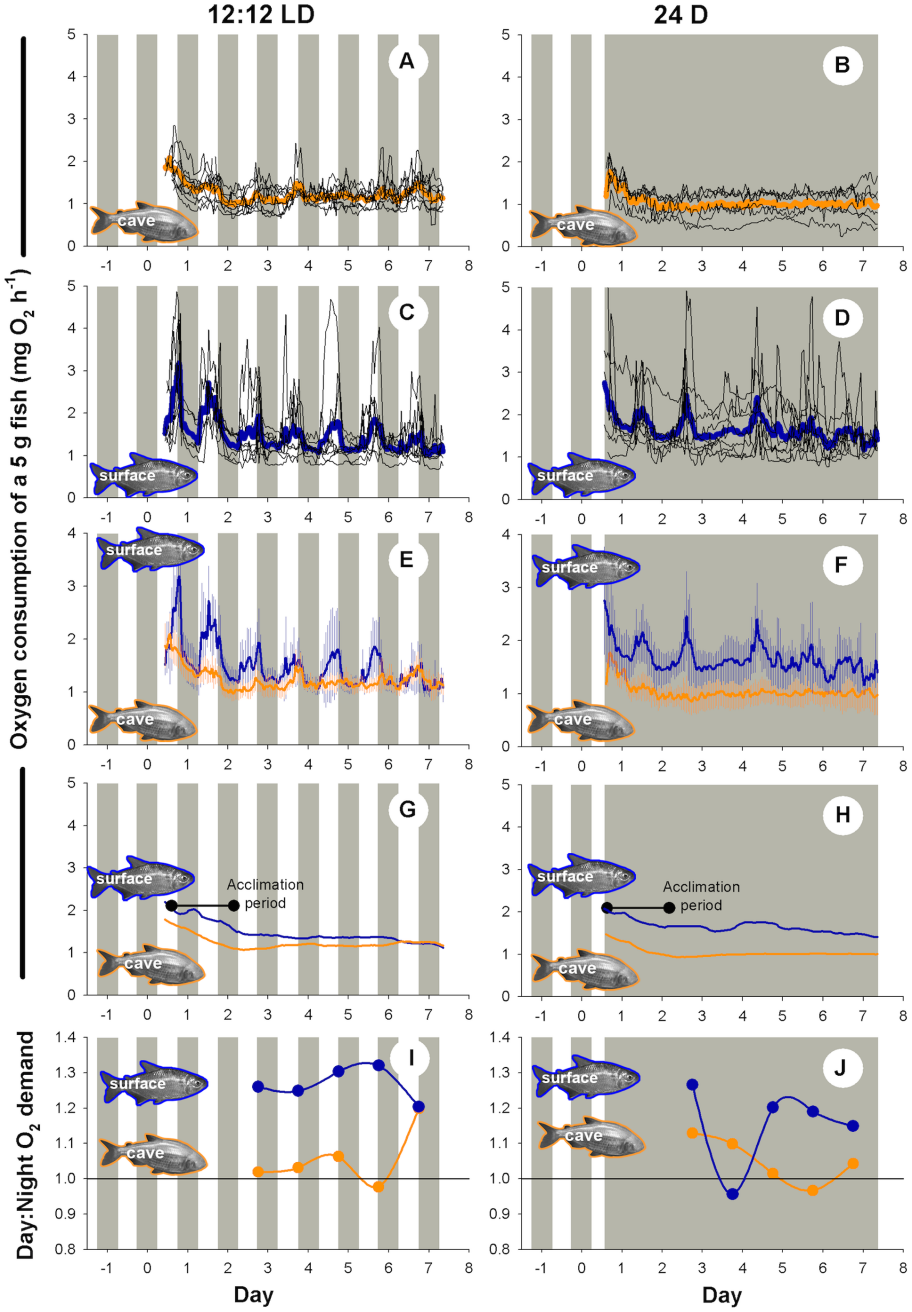
Comparison of the metabolic rate (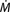
O_2mean_) of *A. mexicanus* over 7 days at a set swimming speed and two photoperiods. (A, C, E, G, I) 12:12 light:dark and (B, D, F, H, J) 24 h dark photoperiod. Grey areas represent dark conditions. A to D display the traces for all individuals with the mean superimposed. E and F display mean and 95% confidence intervals. G and H display 24 h running means, and the arrow indicates when acclimation was deemed complete. I and J display the ratio of daytime:nighttime total oxygen consumption.

To determine whether the metabolic rate of *A. mexicanus* was under circadian clock control we measured the oxygen consumption of surface and Pachón forms under a light/dark cycle and in constant darkness. Our study collected 9401 oxygen consumption measurements from 31 individuals over 7–8 days (equivalent to 2350 hours of recording) at a single swimming speed and two photoperiods (12:12 LD diel photoperiod and 24 D constant darkness). The metabolic rate (time resolved 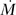
O_2mean_) decreased markedly over the first 1.5 days of respirometry for both fish types as they adapted to respirometry conditions and possibly passed through the last stages of assimilation of their meal 40 h earlier (Figure 2A–D). When mean oxygen consumption over 24 h was plotted it was observed that the metabolic rate had stabilised by the second day of testing (Figure 2G and H). For circadian rhythm comparisons we discarded the first 36 h of data, and only analysed the following 5.5 days of measurements. The considerable decrease in metabolism during the first 36 h of respirometry is important as other respirometric studies of this species have not accounted for adaptation to confinement. The individual variation in oxygen consumption over time was more marked for surface forms compared to cave forms (95% confidence interval considerably larger for surface forms, Figure 2E & F). Surface fish had elevated oxygen consumption during daylight hours under the 12:12 LD photoperiod, although the amplitude of the increase was highly variable between individuals (Figure 2C and E). Comparison of total daytime to nighttime oxygen demand under the 12:12 LD photoperiod indicated that surface forms had a consistently elevated level of metabolism during daytime for the duration of the experiment, while Pachón forms exhibited similar levels of oxygen use day and night, except for the last day of testing (Figure 2I). Under 24 D, the surface forms initially exhibited an elevation in oxygen demand during the subjective daytime, however, on day 3 there was significant damping of the circadian rhythm for most individuals (Figure 2D, 2F and 2J). After this time, surface fish exhibited considerably reduced rhythmicity and synchrony between individuals, although on average they tended to consume approximately 20% more oxygen during the subjective daytime (Figure 2J). Pachón forms did not display a clear pattern of circadian rhythm in metabolism under constant dark conditions (Figure 2F and 2J).

### Minimal metabolic rate

Comparison of 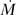
O_2min_ under each morph's natural photoperiod showed no significant difference (Dunn's post hoc test) in minimal metabolism (mean±SD, 0.218±0.043 for surface morphs versus 0.173±0.043 mg O_2_ h^−1^ g^−1^ for Pachón morphs), suggesting that the underlying energetic physiology of both morphs is not substantially different. Comparison of the allometrically scaled 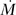
O_2min_ to account for the effect of body size on the metabolic data did not change this finding (Table 1). The two other respirometric studies of these morphs have derived minimal metabolism values from data collected solely under constant dark conditions [11], [13]. Our results show that testing surface fish under constant dark conditions leads to an over-estimation of minimal metabolism by 18% (Table 1), which may be part of the reason why previous studies have noted relatively large differences in minimal metabolism between morphs.

### Effect of photoperiod on oxygen consumption

**Figure 3:**
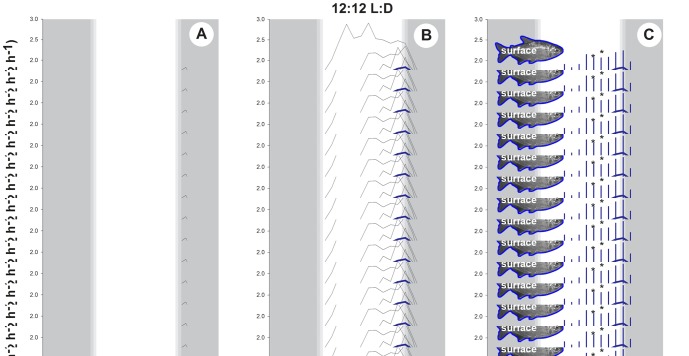
Daily variation in the allometrically scaled metabolic rate (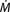
O_2mean_) of *A. mexicanus* at a set swimming speed and two photoperiods. (A–C) a 12:12 light:dark and (D–F) 24 h dark photoperiod. Grey areas represent dark conditions. Each trace represents the combined data for 5.5 days. A, B, D and E display the traces for all individuals with the mean superimposed. C and F display mean with 95% confidence intervals. Asterisks indicate time periods where means differ at *P*<0.05.

While minimal metabolic rate did not differ significantly between morphs, the profile of daily oxygen demand differed considerably. The allometrically scaled 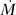
O_2mean_ was used to analyze how the metabolism varied per hour, and surface fish exhibited a pronounced diel variation in metabolism under a 12:12 LD photoperiod (Figure 3B), with peak oxygen consumption typically occurring at a time of day around 17:13 (Table 1). Prior to the artificial sunrise the 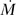
O_2mean_±SD was 1.29±0.20 mg O_2_ h^−1^ 5 g^−1^, after which it increased steadily throughout the day to a maximum of 1.83±0.58 mg O_2_ h^−1^ 5 g^−1^ in the hour before artificial sunset (Figure 3B). After sunset the oxygen consumption rate decreased rapidly over the subsequent three hours to a near-steady nighttime value of approximately 1.30 mg O_2_ h^−1^ 5 g^−1^. The total daily oxygen use of surface fish was significantly higher (by 27%, *t*(7) = 3.006, *P* = 0.020) than the total oxygen consumption at night (Table 1). The profile of daily metabolic rate in Pachón Mexican cavefish under a 12:12 LD photoperiod suggested there was a slight increase in oxygen consumption in the afternoon (Figure 3A) with a similar time of day peak as that of surface fish (16:30, Table 1), however, the metabolic increase was too small to result in any significant difference in daytime versus nighttime oxygen consumption (*t*(8) = 1.514, *P* = 0.168).

There was evidence of a circadian rhythm in surface fish after transfer from a diel photoperiod to constant dark conditions, with a gradual increase in oxygen consumption during the subjective morning and afternoon, followed by a decrease during the subjective dusk to an early morning minima (Figure 3E). The total oxygen consumption of surface fish during the subjective day was significantly higher (by 15%, *t*(7) = 3.360, *P* = 0.012) than the total oxygen consumption during the subjective night (Table 1). The time of day of peak metabolism for surface fish was 11:38, approximately 4.5 h earlier than that recorded under a diel photoperiod. There was a large degree of variation around the peak metabolism times (±4 h, Table 1) limiting the ability to make inferences about the period of the free-running internal clock. The metabolic rate of surface fish under the 24D photoperiod was generally elevated compared to surface fish under the diel photoperiod: values of both 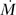
O_2min_ and 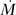
O_2mean_ were elevated, and were significantly higher than equivalent values in Pachón fish (which was not the case under the more natural 12:12 LD photoperiod of the surface fish, Table 1). This suggests that surface fish experience some physiological stress after transfer from diel to constant dark conditions. Pachón fish showed no evidence of a circadian rhythm in metabolism in constant darkness. The oxygen consumption of the cave morph was stable under 24 D (Figure 3D) with no evidence of the late afternoon increase in metabolism that was observed under a light/dark cycle.

### Daily energy expenditure in different photoperiods

As is summarized in Table 1, the effective loss of the metabolic circadian rhythm by Pachón *A. mexicanus* has considerable consequences on daily energy expenditure when compared to surface fish, the latter morph being representative of the ancestral state for this species. The Pachón cave morph spends 27% less energy per day than the surface morph when comparing both under their natural photoperiods (based on the scaled daily energy expenditure). If a surface fish is washed into a cave then it is predicted to spend 16% more energy per day than it did above ground, and over 38% more energy than a Pachón cave fish living in the dark. In summary, the data suggest that the loss of the daily increase in metabolism is the main energetic factor that has changed during the adaptation of *A. mexicanus* to subterranean life, rather than a substantial change in the underlying physiological energetic profile of ancestral surface forms that diversified into caves.

## Discussion

The minimal metabolism measurements made in this study concur with the analysis by Hüppop [12] that there is no statistically significant difference in the resting metabolic rate of Pachón and surface forms of *A. mexicanus*. This result is in contrast with the results of Hüppop's original study [11], and the finding by Salin et al of a 50% reduction in metabolic rate in Pachón morphs [13]. One particular limitation of previous studies is the relatively short measurement times used, as it was clear from our results that fish require 24–36 h to adapt to respirometry conditions. In addition, both the Hüppop and Salin et al studies only measured oxygen consumption in constant darkness. In the present study, the 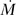
O_2min_ of surface forms in constant darkness was 18% higher compared to measurements made under their natural diel photoperiod. As was noted in our study and by Hüppop [11], surface *A. mexicanus* exhibit a considerable degree of inter-individual variation in metabolic rate and the profile of oxygen demand during the day. The high degree of inter-individual variability in circadian oxygen consumption observed in the current study is characteristic of many fish chronobiology studies [30], and would likely serve as a useful attribute for diversification into caves. Given that there was no significant difference in 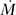
O_2min_ between surface and Pachón fish when tested under natural photoperiod conditions, the differences between morphs in daily energy expenditure observed in the current study are not due to a substantial alteration in the underlying physiological energetics of this species. The eyes of surface fish would not account for the signficant increase in daytime metabolic energy use as the vertebrate retina is more expensive in the dark than the light [37]. Rather, our data suggest that the most important contribution in terms of energy savings for Pachón *A. mexicanus* is a loss of the circadian rhythm in metabolic rate.

Under a diel photoperiod surface fish showed an increased metabolic rate during daytime, a pattern consistent with its lifestyle as a day-active [38] predator and omnivore [39]. The total daytime oxygen demand was approximately 20% higher than at nighttime in surface morphs, which concurs with the magnitude reported for other fish species [21], [26]. In contrast, Pachón fish had a steady metabolic rate when held under the constant dark conditions that simulate a cave. The difference in daily metabolic profiles was not simply a reflection of variation in activity level, as the swim flume respirometer had a constant current velocity and imposed a set swimming speed. Two other studies of fish metabolism using flume respirometers with fixed swimming speeds have observed similar circadian rhythms in oxygen consumption [21], [26]. While activity-controlled metabolic rate studies are scarce (most vertebrates studied to date cannot have their activity level set to a single intensity for seven days), a growing literature on metabolic cycles and disease shows that many aspects of vertebrate energy metabolism follow a circadian rhythm independent of activity [25], [40], [41]. The coupling between molecular clocks and physiology is poorly understood, but there is evidence that for animals with circadian patterns in activity, foraging and digestion, a circadian timing system allows animals to anticipate metabolic reactions during the course of the day in a proactive manner [41]. The circadian increase in metabolic rate in higher vertebrates has been linked to the upregulation of gene expression and enzymes involved in energy metabolism. A large number of enzymes showing circadian gene expression in mammals are involved in mitochondrial oxidative phosphorylation, metabolic fuel mobilisation, and flux through several energy related pathways (lipid synthesis and oxidation, sterol synthesis and hepatic gluconeogenesis) [42]. The loss of a circadian metabolic rhythm by Pachón *A. mexicanus* is likely related to a reduced need to upregulate energy metabolism in anticipation for regular daily increases in activity and feeding.

A common approach to understanding biological rhythms is to remove zeitgebers and record the time-resolved effect on a given trait. In the case of *A. mexicanus*, the removal of light as a zeitgeber to surface fish is not solely a laboratory condition, it also represents a natural condition that ancestral and extant surface fish were and are exposed to [6]. If the metabolic rate of surface fish were under passive control (i.e. no internal oscillator moderating daily energy demand and metabolic rate varied only according to the degree of illumination), then surface fish finding themselves in a cave would be expected to have a daily metabolic rate profile comparable to that of Pachón fish. This scenario was not observed in our results, but rather the surface fish exhibited a circadian rhythm in metabolic rate under constant darkness, with a higher rate during the subjective daytime. Our findings concur with other chronobiology studies of this species, namely that surface fish possess a functional endogenous circadian oscillator, while Pachón morphs have a substantially altered oscillator [20], [43]. It is as yet unclear whether the effective loss of a metabolic and behavioural circadian rhythm in Pachón morphs is due to an altered molecular clock, or a decoupling of these traits from an internal clock or clocks.

The use of automated long-term respirometry and photoperiod testing enabled us to develop novel insights into the relationship between energy metabolism and circadian rhythms in *A. mexicanus*. We were able to confirm that Pachón cave fish have indeed evolved an energy saving strategy that undoubtedly aided their diversification into caves, and our findings suggest a specific mechanism by which this energy saving have been achieved. The energy saving adaptation differs to previously known strategies animals use to cope with food-limited environments such as hypoactivity, dwarfism, cannibalism and growth depression. By eliminating the circadian rhythm in metabolism Pachón fish are able to survive using 27% less daily energy than surface forms in their respective natural lighting conditions. When a surface fish is washed into a cave, our data indicates that the daily metabolic cost for this individual is 16% higher than in a light/dark scenario, presumably due to the stress of finding itself in an alien photoperiod. Under these constant dark conditions, a Pachón *A. mexicanus* will consume around 38% less energy per day than a surface fish flushed into a cave. From this perspective, the energy savings that Pachón fish have achieved by eliminating the circadian rhythm in metabolism are impressive, and possibly explains why this species has not needed to undergo the more radical physiological rearrangements typical of animals that have diversified into food-limited environments. To our knowledge there are no other comparable studies of animals that live in constantly dark habitats, presumably owing to the difficulty in obtaining animals and/or carrying out long-term respirometry with photoperiod testing. However, this would certainly be a worthy line of investigation given the magnitude in energy savings that animals living in perpetual darkness can make by reducing or eliminating the circadian rhythm in metabolism. While it is a strange thought for terrestrial vertebrates to entertain, it may be unnecessary for animals living in caves or the deep sea to rouse their metabolism for the onset of a day that will never arrive. This strategy would certainly be advantageous for animals living in food-limited deep sea or subterranean habitats that are devoid of ciradian periodicity in predators and nutrient availability.
